# Transcranial ultrasound stimulation parameters for neurological diseases: a systematic review

**DOI:** 10.3389/fneur.2025.1567482

**Published:** 2025-05-21

**Authors:** Jingxuan Wang, Hujun Wang, Tingyu Jiang, Yingpeng Wang, Ning Li, Shuyan Qie

**Affiliations:** ^1^Beijing Rehabilitation Medicine, Beijing Rehabilitation Hospital, Capital Medical University, Beijing, China; ^2^Department of Rehabilitation, Beijing Rehabilitation Hospital, Capital Medical University, Beijing, China

**Keywords:** physiotherapy, rehabilitation, non-invasive neuromodulation, transcranial ultrasound stimulation, neurological diseases

## Abstract

**Background:**

Transcranial ultrasound stimulation (TUS) is a non-invasive neuromodulation technique with promising clinical potential. Its therapeutic efficacy and safety are significantly influenced by stimulation parameters. This study investigates how various stimulation parameters modulate human brain function, offering insights for optimizing stimulation protocols to improve clinical and research outcomes.

**Methods:**

A comprehensive literature search was conducted across the Medline-PubMed, Web of Science, Medline-Ovid, Embase, EBSCOhost, Cochrane Library, CNKI, WanFang, and VIP databases using the keyword “transcranial ultrasound stimulation,” covering publications up to September 24, 2024. Two researchers independently screened articles according to predefined inclusion and exclusion criteria. Extracted data included article details, demographic information, interventions, study design, data analysis, and results. The risk of bias was assessed using the RoB2 and ROBINS-I tools. Multiple linear regression analysis explored the relationship between TUS parameters and human physiological responses.

**Results:**

Thirty-five studies were included, consisting of 10 randomized controlled trials and 25 other studies, involving 664 participants (over 34% female) aged 10 to 90 years. Eighteen studies used focused transcranial ultrasound (fTUS), six used non-focused TUS (no-fTUS), and 11 used transcranial pulse stimulation (TPS). Fundamental frequencies ranged from 220 to 650 kHz, and spatial peak pulse average intensities (I_SPPA_) ranged from 0.5 to 31 W/cm^2^. Frequency, pulse repetition frequency, and mechanical index showed significant effects (*p* < 0.05).

**Conclusion:**

TUS demonstrates disease-specific therapeutic potential, with low-frequency protocols targeting neurodegenerative disorders and high-frequency parameters effectively alleviating motor symptoms. Core parameters (fundamental frequency, pulse repetition frequency, mechanical index) modulate neuroplasticity-driven outcomes, accompanied by mild transient adverse effects (incidence < 5%). Large-scale randomized trials integrating multimodal navigation are required to standardize dose-response frameworks and refine spatial targeting for clinical translation.

**Systematic review registration:**

https://www.crd.york.ac.uk/prospero/, identifier: CRD42024601735.

## 1 Introduction

Non-invasive brain stimulation (NIBS) is a widely used neuromodulation technique that modulates neural activity through physical stimuli, offering a safe method with minimal adverse effects (1). In recent years, the expanding clinical use of NIBS has driven research into the factors influencing its efficacy. Among NIBS techniques, transcranial magnetic stimulation (TMS) (2) and transcranial electrical stimulation (TES) (3) have demonstrated effectiveness in stimulating cortical areas and treating neurological and psychiatric conditions. Extensive research, including clinical trials, animal studies, and computational simulations, has explored the parametric paradigms of these techniques (4–7). However, these methods have limitations in targeting deeper brain structures and often lack focal precision (8, 9).

Transcranial ultrasound stimulation (TUS) is an emerging technique that uses ultrasound waves to modulate neuronal activity (10). TUS operates through the mechanical and cavitation effects of ultrasound (11), causing deformation of neuronal cell membranes (12), altering ion channel states (13–15), and regulating neuronal excitability (16). Unlike traditional NIBS methods, TUS can non-invasively stimulate both cortical and deeper brain regions with high precision through incident wave interference (17). The effective depth of TUS can reach 5 to 7 cm below the cerebral cortex (18). Currently, TUS is adaptable to various clinical and research applications, including neurodegenerative diseases (19–28), psychiatric disorders (29–32), and cognitive enhancement (33, 34). As research progresses, TUS has the potential to treat neurological and psychiatric disorders and offering new hope for patients.

Previous systematic reviews have summarized TUS's effects on brain excitability and behavior, offering valuable references for TUS research and clinical application (35–37). Firstly, the number of clinical studies on TUS is limited, with a primary focus on healthy subjects, and there is a lack of systematic research targeting specific patient populations. This restricts the broader application and translation of TUS into clinical practice. Secondly, the relationship between TUS stimulation parameters (such as frequency, intensity, duration, pulse patterns, etc.) and therapeutic outcomes has not been thoroughly explored, and there is a lack of standardized parameter optimization protocols. This has led to poor reproducibility and consistency in research findings. Therefore, understanding how these parameters influence therapeutic effects is crucial for optimizing TUS protocols.

This study aims to conduct a comprehensive systematic review and analysis of clinical studies involving TUS. Specifically, we aim to summarize key parameters such as fundamental frequency, intensity, and pulse characteristics, and analyze their interactions with therapeutic outcomes. This review will provide a reference for the clinical application of TUS and promote its further development in neuroscience and clinical medicine.

## 2 Method

This study adhered to Preferred Reporting Items for Systematic Reviews and Meta-Analyses (PRISMA) guidelines (38) and was registered on PROSPERO (Registration Number: CRD42024601735).

### 2.1 Search strategy

JXW and HJW conducted a comprehensive search of the Medline-PubMed, Web of Science, Medline-Ovid, Embase, EBSCOhost, the Cochrane Library, CNKI, WanFang, and VIP databases for published literature from their inception to September 24, 2024. To maximize study inclusion, we also searched Clinical Trial registries and screened the reference lists of included studies and relevant review articles. The PubMed search strategy is detailed in Table 1, and the full search strategy is available in Supplementary material 1.

**Table 1:** PubMed strategy.

**Step**	**Retrievable**
#1	“neuromodulation”[All Fields]
#2	“transcranial ultrasound”[All Fields] OR “transcranial ultrasound stimulation”[All Fields]
#3	“focused ultrasound”[All Fields] OR “focused ultrasound stimulation”[All Fields]
#4	“transcranial focused ultrasound”[All Fields] OR “transcranial focused ultrasound stimulation“[All Fields]
#5	“transcranial unfocused ultrasound”[All Fields] OR “transcranial unfocused ultrasound stimulation”[All Fields]
#6	“transcranial pulse stimulation”[All Fields]
#7	#2 OR #3 OR #4 OR #5 OR #6
#8	#1 AND #7

### 2.2 Inclusion criteria and exclusion criteria

Studies were included in the systematic review based on the following criteria: (1) Participants included classical neurodegenerative diseases, such as Alzheimer's disease, Parkinson's disease, but also considered other diseases, such as stroke, epilepsy, etc. And there are no restrictions on age, gender, or race; (2) The primary intervention was TUS for the treatment of neurological diseases, including focused ultrasound stimulation, unfocused ultrasound stimulation, and transcranial pulse stimulation, administered in clinical settings by trained health professionals; (3) Eligible study designs included randomized controlled trials, cohort studies, case-control studies, and observational studies that provided details on stimulation parameters; (4) Only published articles were included, and for Chinese studies, only those indexed in the China Social Sciences Citation Index (CSSCI) were considered to ensure quality and credibility.

Exclusion criteria included: (1) Studies involving only healthy individuals; (2) Any form of ultrasound stimulation not specifically targeted at the brain; (3) Studies involving unproven or experimental ultrasound techniques without proper scientific validation; (4) Studies lacking clear descriptions of the intervention or outcomes, case reports with limited data, and animal studies; (5) Studies indexed only in the Engineering Index (EI) or abstracts without full articles; (6) Unfinished experiments registered on clinical trial platforms and research protocols were excluded, as they lack definitive results and may undergo design or implementation changes.

### 2.3 Study selection

Eligible articles were selected following the predefined search strategy. After removing duplicates using EndNote X9, two researchers (JXW and HJW) independently screened the titles and abstracts. The full texts of the remaining articles were then assessed. Disagreements were resolved by a third reviewer (SYQ), and exclusion reasons were documented. There is a good agreement between the two researchers (Kappa = 0.93). The detailed article selection process is illustrated in the PRISMA flow diagram.

### 2.4 Data extraction

The same researchers independently extracted article details, demographic information, interventions, study design, data analysis, and outcomes. For studies with insufficient or unclear data, we contacted the corresponding authors for clarification. Discrepancies between the two researchers were resolved through discussion with a third researcher to reach consensus. The complete data extraction results are presented in Table 2.

**Table 2:** Complete information and data extraction.

**Project**	**Content**
Article information	Title, author, year of publication, country, and contact information of corresponding author
Demographic information	Subject type, age, sex, nationality, sample size, education
Interventions	TUS type, TUS device, target, treatment parameters, and stimulation sessions
Study design and data analysis	Study type, study duration, length of follow-up, data processing method, and statistical methods
Outcomes	Main outcome indicators, evaluation time point of outcome indicators, conclusion, and adverse effects

### 2.5 Risk of bias assessment and studies quality assessment

The risk of bias in the included studies was assessed using appropriate standardized tools. For randomized controlled trials (RCTs), the Cochrane Risk of Bias Assessment Tool 2 (RoB 2) was employed to evaluate potential biases, including selection bias, performance bias, detection bias, attrition bias, reporting bias, and other biases (39). For non-randomized controlled trials (nRCTs), the Risk of Bias in Non-randomized Studies of Interventions (ROBINS-I) tool was used (40). This assessment addressed bias due to confounding, participant selection, intervention classification, deviations from intended interventions, missing data, outcome measurement, and selective reporting. Two researchers (JXW and HJW) independently conducted the assessments. In cases of disagreement, a third researcher (SYQ) facilitated discussions to resolve discrepancies. The risk of bias for each study was categorized as low risk, unclear risk, or high risk.

### 2.6 Strategy for qualitative synthesis

The following strategies for qualitative data synthesis were employed:

1) Basic study information: for each combination of ultrasound type and disease type, relevant data were extracted, including sample size, sex, age, average outcome measures, and study design. Basic descriptive statistics were conducted for each group to summarize the data.
2) TUS type and device details: detailed information about the TUS devices was extracted, including the manufacturer, device type, fundamental frequency, target area, sensor type, shaving requirement, and gel type used during procedures.
3) Same type of TUS in different disease groups: TUS parameters were extracted and analyzed across different disease groups. These parameters included the target area, fundamental frequency, pressure, intensity, timing, and other relevant settings such as pulse duration (PD), pulse repetition frequency (PRF), duty cycle (DC), sonication duration (SD), and mechanical index (MI). The TUS parameters varied depending on the specific disease being treated.
4) Different types of TUS within the same disease group: for each disease group, various TUS parameters were examined in conjunction with the observed treatment effects. Variations in stimulation targets and parameter settings influenced both the extent of treatment effects and the range of symptom improvement.
5) Stimulation targets: studies with different TUS stimulation targets were identified. For each stimulation target, TUS parameters were summarized to evaluate how different ultrasound settings influenced outcomes in various diseases.

### 2.7 Statistical analysis

This study utilized multiple linear regression analysis to explore the relationship between different parameters of transcranial focused ultrasound stimulation (tFUS) and human physiological responses. The independent variables included frequency, peak negative pressure, spatial-peak pulse-average intensity, pulse repetition frequency, duty cycle, pulse duration, and mechanical index, while the dependent variable was the standardized mean difference (SMD) calculated for each study.

SMD calculations were performed by comparing mean values and standard deviations reported for experimental and control groups, and if the studies were before-and-after comparisons within the same subjects, the measurements before and after ultrasound stimulation were used. The formula for combined standard deviation was applied as follows:


$$\begin{array}{l} SD_{\text{combined}}=\sqrt{\frac{\left(n_{\exp}-1\right)SD_{\exp}^{2}+\left(n_{\text{ctrl}}-1\right)SD_{\text{ctrl}}^{2}}{n_{\exp}+n_{\text{ctrl}}-2}} \end{array}$$

To account for potential biases due to small sample sizes, SMD was adjusted using Hedges' g correction:


$$\begin{array}{l} g=\text{SMD}\times \left(1-\frac{3}{4N-9}\right) \end{array}$$

where *N* is the total sample size of the experimental and control groups. For studies that did not report mean and standard deviations, effect sizes were estimated from *t*-values or F-values using:


$$\begin{array}{l} \text{SMD}=\frac{t}{\sqrt{n_{\exp}+n_{\text{ctrl}}}}\;or\;SMD=\sqrt{\frac{F}{n_{\exp}+n_{\text{ctrl}}}} \end{array}$$

and from correlation coefficients as:


$$\begin{array}{l} SMD=\frac{2r}{\sqrt{1-r^{2}}} \end{array}$$

In cases of incomplete data, efforts were made to obtain additional information through communication with corresponding authors or such studies were excluded from the regression analysis.

Regression model construction involved ordinary least squares (OLS) to formulate the multiple linear regression model, represented as:


$$\begin{array}{l} y=\beta_{0}+\beta_{1}\cdot FF+\beta_{2}\cdot PNP+\beta_{3}\cdot ISPPA+\beta_{4}\cdot PRF+\beta_{5}\cdot DC\\ \;+\beta_{6}\cdot PD+\beta_{7}\cdot MI+\epsilon \end{array}$$

Model fit was assessed through *R*^2^ and adjusted *R*^2^, and the significance of regression coefficients was evaluated via t-statistics and *p*-values, facilitating the identification of parameters with independent effects on the effect sizes.

## 3 Result

### 3.1 Study selection

**Figure 1:**
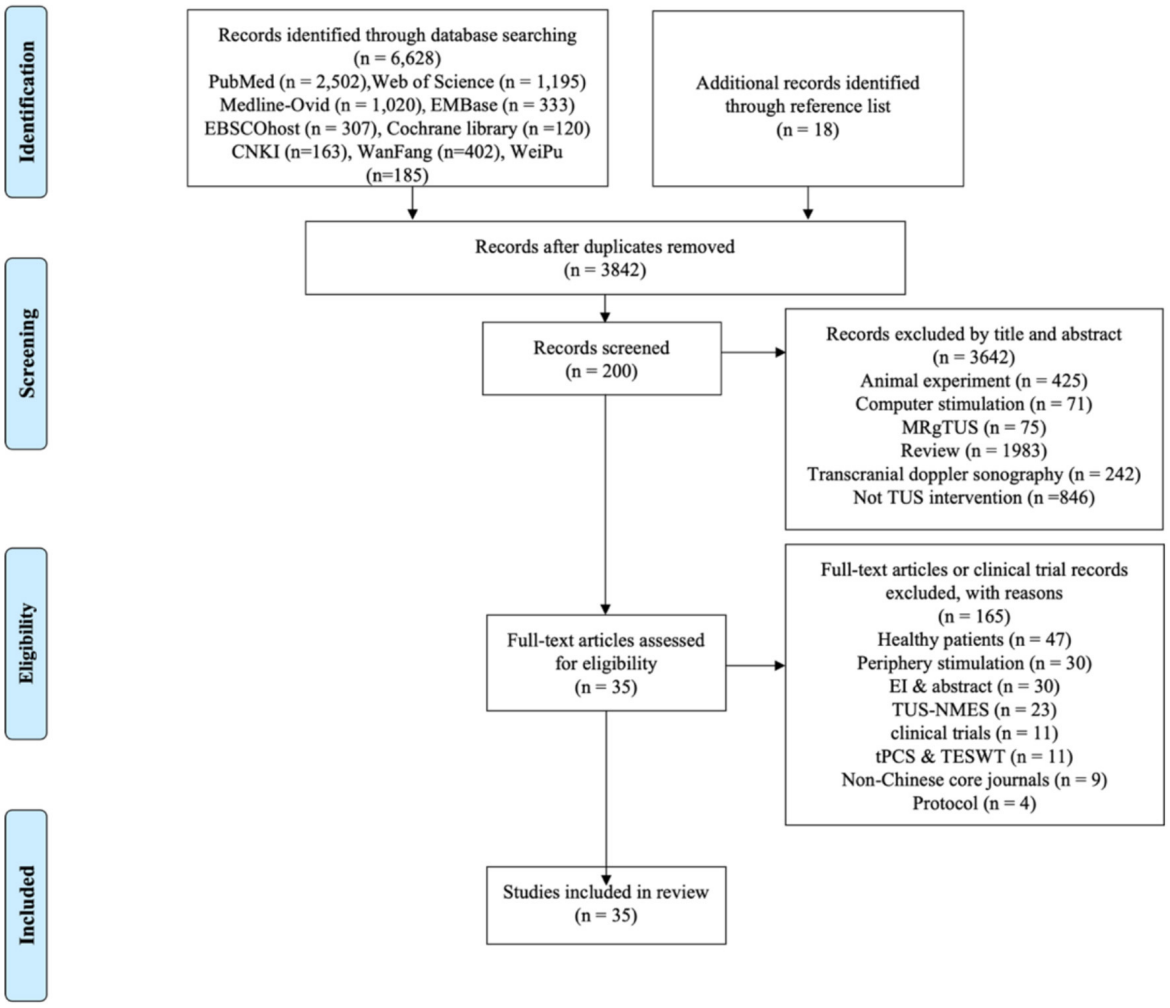
Selection process and reasons for exclusion PRISMA flow chart.

A total of 6,646 studies were identified through the search strategy. After removing duplicates, 3,842 studies remained. Following title and abstract screening, 200 studies were selected for full-text review. Finally, after applying exclusion criteria, 35 studies met the eligibility criteria for inclusion in the systematic review. The selection process and reasons for exclusion are illustrated in the PRISMA flow chart (Figure 1).

### 3.2 Risk of bias assessment

**Figure 2:**
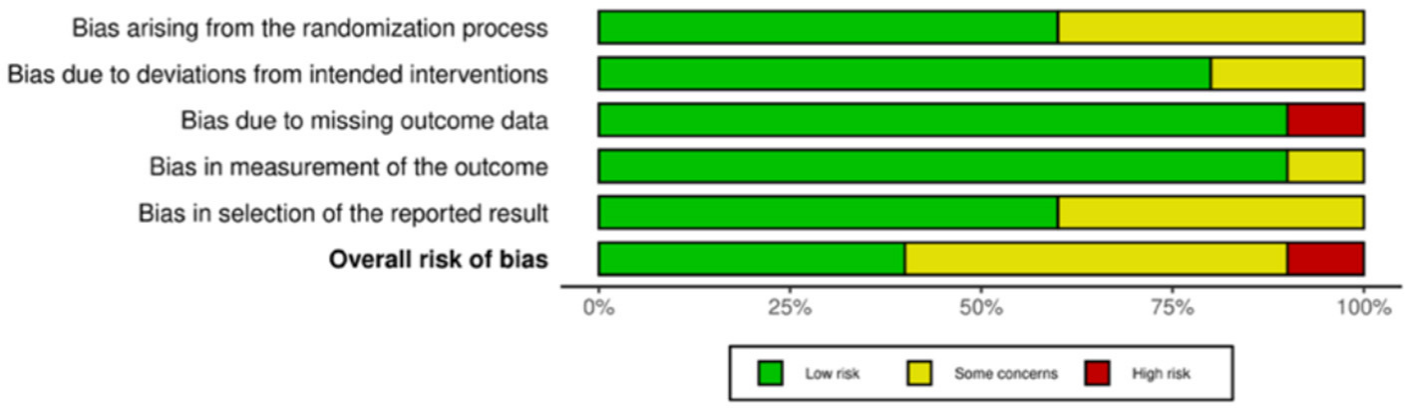
Risk of bias summary in RCT studies.

**Figure 3:**
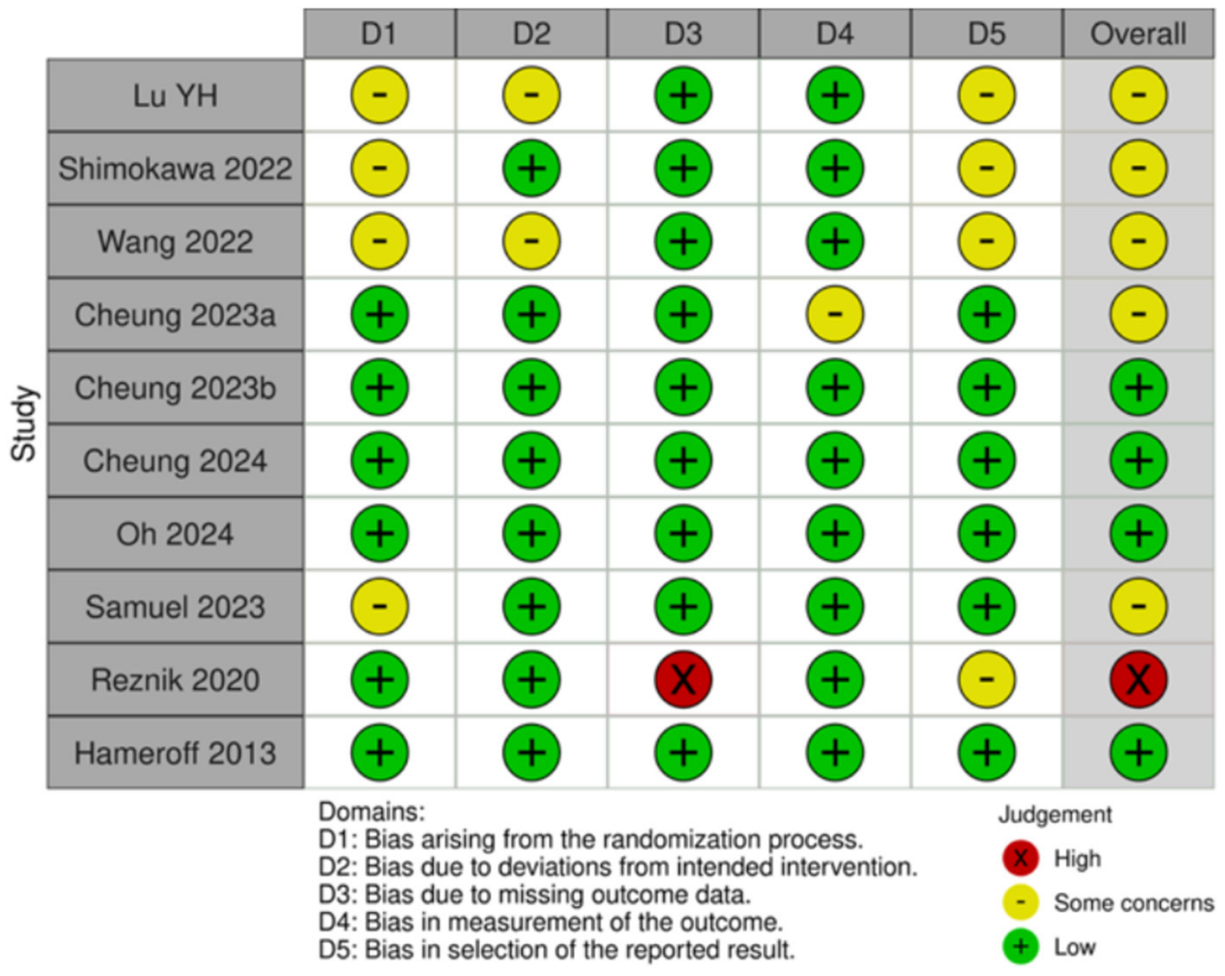
Risk of bias domains in RCT studies.

**Figure 4:**
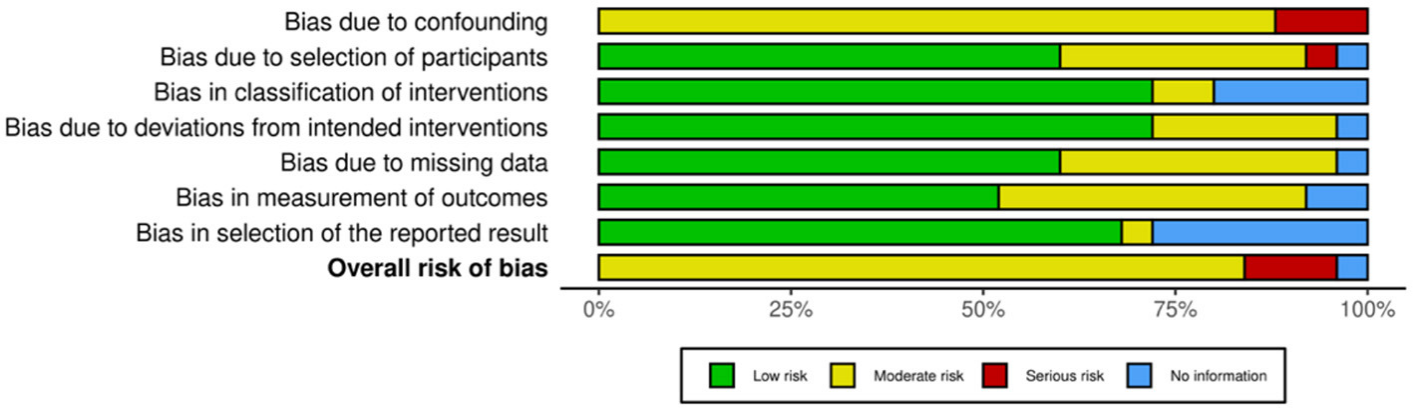
Risk of bias summary in nRCT studies.

**Figure 5:**
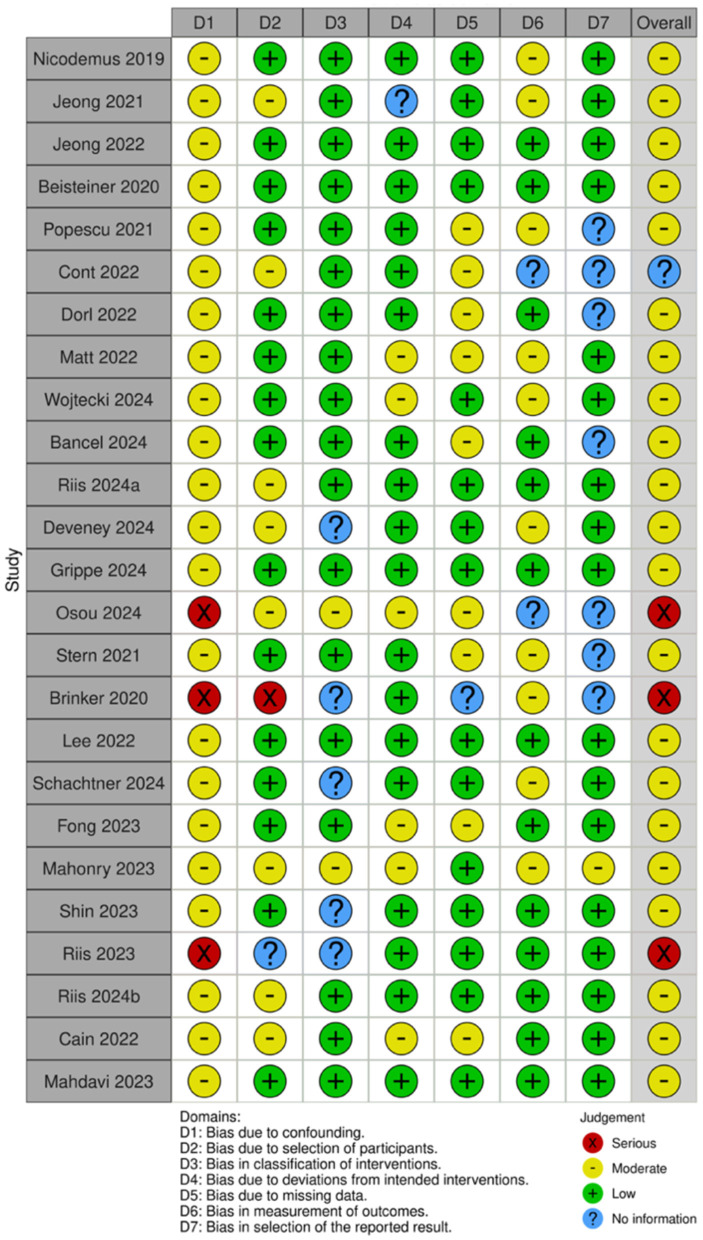
Risk of bias domains in nRCT studies.

Of the 35 included studies, 10 were RCTs (22, 29–31, 41–46) and 25 were nRCTs (19–21, 23–28, 32–34, 47–59). According to the Cochrane Handbook for Systematic Reviews, the RoB 2 tool was used to assess the RCTs. The bias assessment revealed that four studies had a low overall risk of bias (31, 42, 43, 45), five studies had a moderate risk (22, 30, 41, 46), and one study had a high risk (29). The results for the RCTs are shown in Figures 2, 3. For the nRCTs, the ROBINS-I tool was applied. The assessment showed that 21 studies had a moderate overall risk of bias (19–21, 23, 24, 26–28, 32–34, 47–49, 51, 52, 54, 56–59), three studies had a high risk (50, 53, 55), and one study had insufficient information (25). The results for the nRCTs are presented in Figures 4, 5.

### 3.3 Demographic information

A total of 664 patients were enrolled in the study, of whom more than 34% were female. The patients' ages ranged from 10 to 90 years. The types of fTUS used in the studies were categorized into three groups: focused transcranial ultrasound (fTUS), non-focused transcranial ultrasound (no-fTUS), and transcranial pulse stimulation (TPS). The diseases investigated in the study were classified into five categories: neurodegenerative diseases, movement disorders, mental disorders, other central nervous system diseases, and pain disorders. Detailed characteristics of the 35 included studies are presented in Table 3.

**Table 3:** Detailed features included in the study.

**References**	**Demographic**			**Intervention**		**Outcome**	**Study design**
	**Patients**	**Sample (Female)**	**Mean age**	**Type**	**Control**		
**1) Neurodegenerative disease**							
Nicodemus et al. (19)	AD/PD	11 (3) /11 (3)	40 ~ 90 y	dUS	NR	CDR; RBANS; MoCA; T25-FW; 9-HPT; MRI	Open label
Jeong et al. (20)	AD	4 (3)	78.88 ± 3.3	fTUS	NR	PET; COWAT; CWST; MMSE; SVLT	Open label
Jeong et al. (21)	AD	8 (7)	78.1 ± 2.9	fTUS	NR	PET; COWAT; CWST; MMSE; SVLT	Open label
Shimokawa et al. (22)	AD	Verum:11 (6) /Placebo: 8 (5)	70.4 ± 3.0 /73.6 ± 3.9	LIPUS	Placebo	ADAS-Jcog; MRI	RCT
Popescu et al. (23)	AD	17	NR	TPS	NR	cortical thickness; CERAD	Single-group pretest-posttest study
Beisteiner et al. (24)	AD	35	NR	TPS	NR	CERAD; CTs; PCA; SEG scale; MRI	Multicenter, non-controlled pilot study
Cont et al. (25)	AD	11 (2)	69.82	TPS	NR	ADAS; MMSE; MoCA; NRS	Retrospective study
Dörl et al. (26)	AD	18 (11)	69.94	TPS	NR	GE; CERAD	Open-label, Follow-up
Matt et al. (27)	AD	18	NR	TPS	NR	BDI- II; MRI	Open-label, Follow-up
Wojtecki et al. (28)	AD	10 (2)	69.2 ± 7.1	TPS	NR	EEG	Open-label
Samuel et al. (44)	PD	10 (2)	63.80 ± 7.15	tbTUS	Sham	MEP; SICI; SICF; MDS-UPDRS- III	Open-label
Osou et al. (53)	PD	20	NR	TPS	NR	UPDRS- III	Open-label, retrospective
Grippe et al. (52)	PD/HC	20 (5) /17 (5)	59.1 ± 8.7 /63.7 ± 9.5	tbTUS	HC	MEP; SICI; SICF; MDS-UPDRS- III	Case-control study design
**2) Movement disorder disease**							
Bancel et al. (48)	ET	9 (2)	65.67 ± 10.21	fTUS	NR	CRST; 3DMR	Open-label
Deveney et al. (47)	ET	10 (4)	73.6 ± 6.42	fTUS	NR	GRC; TETRAS	Open-label
Riis et al. (51)	ET	3 (0)	64.67 ± 16.92	fTUS	Sham	Tremor amplitude	Open-label
**3) Psychiatric and psychological disease**							
Reznik et al. (29)	MDD	24 (16)	18.9 ± 1.1	fTUS	Placebo	VAMS; BDI-II; OASIS	RCT
Cheung et al. (30)	MDD	Verum:15 /Sham: 15	38.8 ± 15.0 /38.8 ± 15.0	TPS	WC group	HDRS-17	RCT
Oh et al. (31)	MDD	Verum: 11 (6) /Sham: 12 (7)	14.3 ± 1.7 /15.3 ± 3.1	fTUS	Sham	MADRS	RCT
Schachtner et al. (32)	MDD	20 (15)	30.4 ± 10.0	fTUS	NR	BDI-II; PTQ; HDRS; WHO QOL; CSSRS	Open-label
Cheung et al. (42)	ASD	Verum: 16 (3) /Sham: 16 (2)	13.5 ± 2.03 /12.81 ± 1.83	TPS	Sham	CARS	RCT
Cheung et al. (43)	ADHD	Verum: 17 (4) /Sham: 15 (3)	12.8 ± 1.51 /13.3 ± 1.34	TPS	Sham	SNAP-IV; CGI; Stroop test; ADHD RS-IV	RCT
Mahdavi et al. (59)	trGAD	25 (11)	38.96 ± 12.61	fTUS	NR	HAMA; BAI; PGI-I	Open-label
Riis et al. (50)	TRD	1 (1)	30	fTUS	NR	BOLD-fMRI; HDRS-6	Open-label
Riis et al. (49)	TRD	2 (2)	32, 35	fTUS	Sham	7-point scale	Open-label
Mahoney et al. (57)	SUD	Group 1: 2 (0) /Group 2: 2 (1)	36.5 ± 3.54 /32.0 ± 2.8	fTUS	Sham	VAS	Open-label
**4) Other Central nervous system disease**							
Lu (46)	Stroke	Verum: 45 (20) /Sham: 45 (21)	63.5 ± 4.9 /64.3 ± 4.2	dUS	Sham	VAS; FMA; BI; ADL	RCT
Wang et al. (41)	Stroke	Active: 30 (7) /Sham: 30 (5)	57.73 ± 7.90 /54.57 ± 4.94	fTUS	Sham	MMSE; MoCA; MBI; P300 latency and amplitude; BDNF	RCT
Brinker et al. (55)	DRE	1 (1)	36	fTUS	NR	ultrasound pressure field data	Case study
Stern et al. (54)	DRE	8 (5)	35.6 ± 14.5	fTUS	NR	fMRI (BOLD); EEG; RAVLT	Prospective study
Lee et al. (56)	DRE	6 (2)	33.0 ± 6.8	fTUS	NR	fMRI; Epileptic Seizure Frequency; IEDs	Open-label
Cain et al. (34)	DOC	11 (2)	45.73 ± 20.43	fTUS	NR	BOLD-fMRI; CRS-R	Open-label
Fong et al. (33)	M-NCD	19 (12)	74.32	TPS	TAU group	HK-MoCA; 30 s; Stroop; BDNF	Open-label, uncontrolled
**5) Pain disease**							
Hameroff et al. (45)	Chronic pain	Verum: 14 (8) /Sham: 17 (11)	53.8 ± 14.7	dUS	Placebo	NRS; VAMS	RCT
Shin et al. (58)	Chronic neuropathic pain	11 (5)	60.55 ± 13.18	fTUS	NR	VAS	Open-label

NR, not report; fTUS, focused Transcranial Ultrasound Stimulation; dUS, diagnostic Ultrasound Stimulation; tbTUS, theta-burst Transcranial Ultrasound Stimulation; LIPUS, Low-intensity Pulsed Ultrasound Stimulation and TPS, Transcranial Pulse Stimulation; CDR, Clinical Dementia Rating; RBANS, Repeatable Battery for the Assessment of Neuropsychological Status; MoCA, Montreal Cognitive Assessment; MMSE, Mini-Mental State Examination; SVLT, Semantic Verbal Fluency Test; CERAD, Consortium to Establish a Registry for Alzheimer's Disease; ADAS-Jcog, Alzheimer's Disease Assessment Scale-Japanese version; ADAS, Alzheimer's Disease Assessment Scale; COWAT, Controlled Oral Word Association Test; CWST, Color-Word Stroop Test; MDSUPDRS-III, Movement Disorder Society-Unified Parkinson's Disease Rating Scale-Part III; CRST, Clinical Rating Scale for Tremor; T25-FW, 25-Foot Walk Test; TETRA, Tremor Rating Scale; FMA, Fugl-Meyer Assessment; BDI-II, Beck Depression Inventory-II; VAMS, Visual Analog Mood Scale; HDRS-17, Hamilton Depression Rating Scale-17; MADRS, Montgomery-Asberg Depression Rating Scale; HDRS-6, Hamilton Depression Rating Scale-6; HAMA, Hamilton Anxiety Scale; BAI, Beck Anxiety Inventory; PET, Positron Emission Tomography; EEG, Electroencephalogram; MEP, Motor Evoked Potential; fMRI (BOLD), Functional Magnetic Resonance Imaging (Blood-Oxygen-Level-Dependent); 3DMR,3-Dimensional Magnetic Resonance; IEDs, Interictal Epileptiform Discharges; 9-HPT, 9-Hole Peg Test; NRS, Numeric Rating Scale; GE, General Evaluation; SICI, Short-Interval Intracortical Inhibition; SICF, Short-Interval Cortical Facilitation; GRC, General Rehabilitation Condition; WHO QOL, World Health Organization Quality of Life; CARS, Childhood Autism Rating Scale; SNAP-IV, Swanson, Nolan, and Pelham, Version IV; CGI, Clinical Global Impression; ADHD RS-IV, Attention-Deficit/Hyperactivity Disorder Rating Scale-Version IV; PGI-I, Patient Global Impression of Improvement; VAS, Visual Analog Scale; BI, Barthel Index; ADL, Activities of Daily Living; BDNF, Brain-Derived Neurotrophic Factor; RAVLT, Rey Auditory Verbal Learning Test; CRS-R, Coma Recovery Scale-Revised; OASIS, Overall Anxiety Severity and Impairment Scale; CSSRS, Columbia Suicide Severity Rating Scale; PTQ, Perseverative Thinking Questionnaire.

### 3.4 TUS parameters

In the included studies, 18 studies employed fTUS, six studies utilized no-fTUS, and 11 studies used TPS. The specific ultrasound parameters are detailed in Tables 4–6.

**Table 4:** Specific information of fTUS parameters.

**Disease**	**Target**	**Fundamental frequency**	**Pressure and Intensity**			**Other parameters**						**Stimulation sessions**	**Outcome**	**References**
			**PNP (MPa)**	**I**_SPPA_ **(W/cm**^2^**)**	**I**_SPTA_ **(W/cm**^2^**)**	**PD (ms)**	**PRF (Hz)**	**DC**	**SD**	**ISI**	**MI**			
AD	Hippocampus	250 kHz	0.135	0.5 ~ 3	0.02 ~ 0.12	20	2	4%	180 s	NR	0.27	NR	Memory, executive function, and cognitive mild improvement	(20)
			NR	NR	NR	20	2	4%	180 s	NR	0.33 ~ 0.88	NR		(21)
ET	Thalamus	650 kHz	0.8	19.8	NR	<30	10~67	5 ~ 30%	5 s	10 s	NR	Different for different patients	Tremor power significantly reduced	(48)
	A-MTL		0.72	NR	NR	10	10	10%	15 s	NR	NR	90 min, 23 trials	Tremor amplitude reduced	(51)
	Thalamus		0.61	14.39	7.1973	5	10	5%	30 s	30 s	0.75	8/w	GRC, TETRAS, ADL: improvement	(47)
MDD	DLPFC	250 kHz	0.3	3	0.56 ~ 2.03	1	5	50%	300 ms	6 s	0.27	3/w; 2 w; 20 min	MADRS: improvement; depression decreased	(31)
	AMPFC	400 kHZ	0.82	NR	0.67	5	10	5%	NR	NR	NR	5/w; 3 w	BDI-II, HDRS and PTQ: improvement; Wellbeing and environmental satisfaction: improvement, but social satisfaction not.	(32)
	FTC	500 kHz	0.65	14	0.071	2	40	8%	30 s	NR	0.9	1/d, 5 d	PSWQ: reduced; overall emotion: improvement; depression decreased	(29)
trGAD	R-amygdala	650 kHz	0.61	14.39	7.1973	5	10	5%	30 s	30 s	0.75	8 w; 10 min	HAMA and BAI: decreased	(59)
TRD	SCC, ACG	650 kHz	1	<190	<0.72	30	NR	0.80%	30 ms	4 s	NR	10 times (each target area), for three target areas	HDRS-6 scores: decreased	(50)
	SGC, VS		1	31	0.233	30	NR	0.75%	30 ms	4 s	1.2	during the two medical visits	depression and anxiety indicators: significant improvement	(49)
SUD	Nac	220 kHz	NR	55, 80	NR	100	NR	3.3%	10 min	900 ms	NR	Sham (First) + LIFU	desire for several substances reduced	(57)
DOC	Thalamus	650 kHz	NR	14.39	0.7197	0.5	100	5%	30 s	30 s	NR	1 or 2 times	CRS-R improvement	(34)
Stroke	FC	NR	NR	1.75	NR	NR	NR	NR	20 min	NR	NR	6/w; 5 w; 20 min	MMSE, MoCA, MBI, P300 latency and amplitude, BDNF: improvement; cognitive improvement	(41)
Chronic neuropathic pain	ACC	250 kHz	<0.95	NR	<0.72	5 ~10	70 ~100	50 ~ 70%	NR	NR	<0.9	3/w; 2 w; 30 min	VAS: improvement; pain improvement	(58)
DRE	SOZs	NR	NR	<2.8	NR	3	100	30%	600 s	NR	0.75	10 min	2 frequencies of epileptic seizures reduced	(56)
DRE	MTL	650 Hz	NR	NR	> 0.72	0.2	250	50%	0.5 s	NR	NR	at least once	RAVLT: slight decline	(54)
					5.76	0.5	100	5%	30 s					
DRE	Hippocampus	548 Hz	0.36	NR	0.5 ~ 2.25	0.72 ~1.00	500	36 ~ 50%	0.5s	7s	NR	Bimonthly treatment within 3 weeks.	NR	(55)

**Table 5:** Specific information of n-fTUS parameters.

**Disease**	**Target**	**Fundamental frequency**	**Pressure and intensity**			**Other parameters**						**Stimulation sessions**	**Outcome**	**References**
			**PNP (MPa)**	**I**_SPPA_ **(W/cm**^2^**)**	**I**_SPTA_ **(W/cm**^2^**)**	**PD (ms)**	**PRF (Hz)**	**DC**	**SD**	**ISI**	**MI**			
**dUS**														
AD	Hippocampus	2 MHz	NR	NR	0.52	NR	NR	NR	1 h	NR	NR	1/w; 8 w; 1 h	62.5% patients' cognitive improvement and 87% patients' motor improvement	(19)
PD	Substantia nigra													
Chronic pain	PFC	8 MHz	NR	NR	0.152	NR	NR	NR	15 s	NR	0.7	2 times	GA and NAS: improvement; Mood improvement	(45)
Stroke	Frontal windows	800 kHz (± 80)	NR	NR	0.75	10 s	6.67 ~ 16.67	100%	NR	NR	NR	2/d; 4 w; 15 min	VAS, FMA, BI: improvement	(46)
**LIPUS**														
AD	Whole Brain	500 kHz	1.3	NR	NR	0.064	781	5%	20 min	1.28 ms	NR	3/d; 3 d; 20 min	Suppress cognitive impairment	(22)
**tbTUS**														
PD	Motor cortex	500 kHz	NR	2.26	0.23	20	5	10%	80 s	30 min	NR	during each visit	Excitability of the motor cortex improvement, UPDRS- III: no change	(44)
			NR	20	NR	20	5	10%	80 s	200 ms	NR	two different dates (> 72 h)	MDS-UPDRS- III the medication group was significantly reduced	(52)

**Table 6:** Specific information of TPS parameters.

**Disease**	**target**	**EL (mJ/mm^2^)**	**FF**	**Pulse**	**Stimulation sessions**	**Outcome**	**References**
AD	DLPFC, MLA/whole brain	NR	1 ~ 5 Hz	6,000	Center 1:6,000 × 4(3/w,4w)/Center 2: 6,000 × 2(2 w)	Memory and cognitive improvement	(24)
	B-FC, B-PC, E-PC	NR	5 Hz	NR	Each ROI is stimulated twice per session; 4 w	Reduce cortical atrophy	(23)
	B-FC, B-LPC, E-PC, B-TC	0.2	4 Hz	6,000	6 (6,000 pulses) or 12 (3,000 pulses) per day/2weeks	ADAS and ADAS cognitive scores improvement	(25)
	LFC, B-LPC, E-PC	0.2	5 Hz	6,000	3/w; 4 w	Visual construction ability decreased	(26)
	B-FC, B-PC, E-PC	0.2 ~ 0.25	5 Hz	6,000	3/w; 4 w	Depression decreased	(27)
	B-FC, B-LPC, E-PC, B-TC	0.2	4 Hz	NR	Different for different patients	Cognitive improvement	(28)
PD	Motor network	0.25	4 Hz	4,000	2/d; 5/w; 2 w	UPDRS- III reduced	(53)
MDD	DLPFC	0.2 ~ 0.25	3 ~ 4 Hz	300	3/w; 2 w; 30 min	BDI: improvement; Depressive symptoms improvement	(30)
ASD	TPJ	0.2 ~ 0.25	2 ~ 4 Hz	800	6/w; 2 w	CARS and CGI improvement	(42)
ADHD	DLPFC	0.25	4 5 Hz	800	3/w; 2 w; 30 min	SNAP-IV: improvement	(43)
M-NCD	Whole brain	0.2 ~ 0.25	4 ~ 5 Hz	6,000	3/w; 2 w	Cognitive improvement; Stroop improvement	(33)

#### 3.4.1 Fundamental frequency

A total of 16 fTUS studies reported the fundamental frequency parameter, ranging from 220 to 650 kHz (20, 21, 29, 31, 32, 34, 47–51, 57–59), while two studies did not specify this information (41, 56). Among the unfocused ultrasound stimulation studies, three studies employing diagnostic ultrasound (dUS) utilized fundamental frequencies between 2.0 and 8.0 MHz (19, 45, 46). Additionally, two transcranial burst TUS (tbTUS) studies and one low-intensity pulsed ultrasound stimulation (LIPUS) study reported a fundamental frequency of 500 kHz (22, 44, 52). Finally, 11 TPS studies utilized frequencies within the range of 4 to 5 Hz (23–28, 30, 33, 42, 43, 53).

#### 3.4.2 Intensity or energy level

The I_SPPA_ of fTUS ranged from 0.5 to 31 W/cm^2^, with variations depending on the treated disease and the targeted brain region. In depression-related studies, I_SPPA_ values ranged from 3.0 to 14.39 W/cm^2^, while in neurological diseases, values were below 3 W/cm^2^. When targeting different brain regions, I_SPPA_ values were below 2.8 W/cm^2^ for cortical areas and ranged from 14.9 to 80 W/cm^2^ for deep brain areas. For no-fTUS, the I_SPPA_ was < 2.26 W/cm^2^. The energy level (EL) for TPS ranged from 0.2 to 0.25 mJ/mm^2^.

### 3.5 Disease

Thirteen studies on neurodegenerative diseases, including Alzheimer's disease (AD) (19–28) and Parkinson's disease (PD) (19, 44, 52, 53), found improved cognitive function in patients. Three studies on movement disorders, specifically essential tremor (ET) (47, 48, 51), showed significant improvements in tremor symptoms and the ability to perform daily activities.

**Figure 6:**
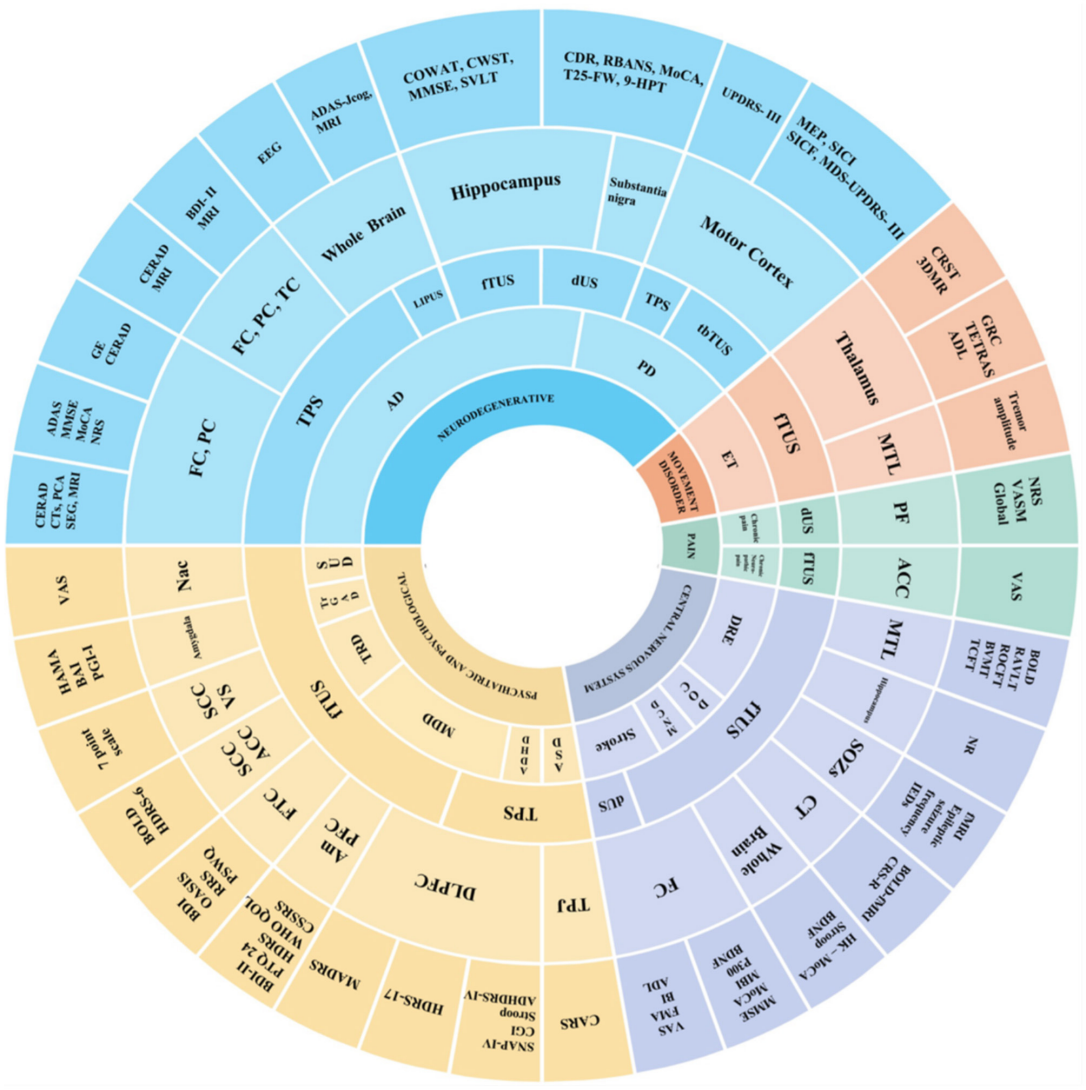
Sunburst chart for main targets and effects of TUS in disease. This sunburst chart depicts the main targets and effects of TUS in diseases. In the chart, different colors signify different types of diseases: Blue (neurodegenerative); Yellow (psychiatric and psychological); Purple (central nervous system); Red (movement disorder); Green (pain). In terms of the circular levels, the inner circle is annotated with diseases, the middle circle successively represents the types of TUS and stimulation target sites, and the outer circle indicates the outcome measures of different studies.

Ten studies on mental disorders—including major depressive disorder (MDD) (29–32), treatment-resistant depression (TRD) (49, 50), treatment-resistant generalized anxiety disorder (trGAD) (59), autism spectrum disorder (ASD) (42), attention deficit hyperactivity disorder (ADHD) (43), and substance use disorder (SUD) (57), found that different parameter combinations could effectively improve depressive and anxiety symptoms. Seven studies on other central nervous system diseases—including stoke (41, 46), drug-resistant epilepsy (DRE) (54–56), mild neurocognitive disorder (M-NCD) (33), and chronic disorders of consciousness (DOC) (34)—reported improvements in cognitive and epileptic outcomes. Studies on pain disorders—chronic pain (45) and chronic neuropathic pain (58)—demonstrated that different parameter combinations could effectively reduce symptoms. Detailed characteristics of the studies are shown in Figure 6.

### 3.6 Targets

In the included studies, TUS was used to modulate different brain regions. The stimulated areas can be divided into two categories: the cerebral cortex and deep brain regions. Twenty-one studies focused on cortical regions, including the motor cortex, frontal cortex, parietal cortex, precuneus cortex, temporal cortex, insula and left. Twelve studies targeted deep brain regions, such as the thalamus, hippocampus, anterior cingulate cortex, subgenual cingulate cortex, amygdala, substantia nigra, ventral striatum, and nucleus accumbens. Two studies focused on the whole brain.

**Figure 7:**
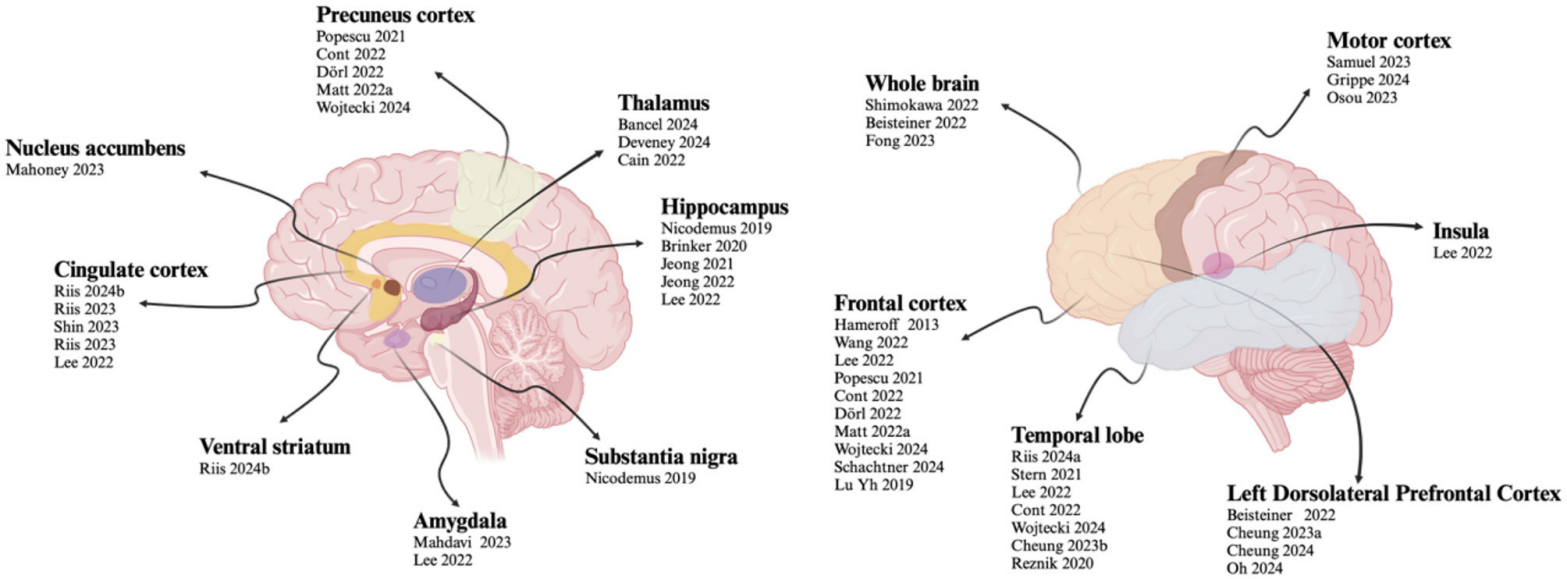
Specific information of TUS target.

**Figure 8:**
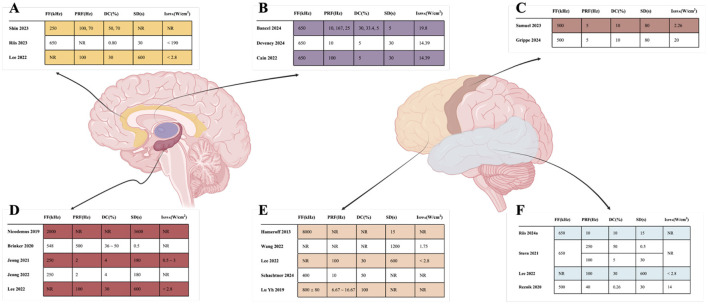
Detailed parameters of TUS target. **(A)** indicated that Cingulate cortex; **(B)** indicated that Thalamus; **(C)** indicated that Motor cortex; **(D)** indicated that Hippocampus; **(E)** indicated that Frontal cortex; **(F)** indicated that Temporal.

For deep brain regions, the TUS parameters were as follows: frequencies ranged from 220 to 650 kHz; pulse durations ranged from <30 ms to 100 ms; pulse repetition rates ranged from 10 to 100 Hz; duty cycles ranged from 3.3 to 70 %; and spatial peak pulse-average intensities I_SPPA_ ranged from 14.39 to 80 W/cm^2^. For cortical regions, frequencies ranged from 500 to 650 kHz; pulse durations ranged from 0.2 ms to 10 ms; pulse repetition rates ranged from 5 to 250 Hz; duty cycles ranged from 5 to 50 %; and I_SPPA_ values were below 2.8 W/cm^2^. The specific detailed parameters are shown in Figures 7, 8.

### 3.7 Other influencing factors

Thirty-three studies reported on localization and navigation methods (19–21, 23–34, 42–59). MRI-based navigation was commonly used; 13 studies employed MRI, including eight that combined it with other techniques such as CT, AntNeuro Visor^2TM^, and EEG. Three studies investigated neuronavigation methods, and two studies used TMS methods. Transducers play a crucial role in ultrasound stimulation devices, and a wide variety of transducers were used in the studies.

Seven studies reported on hair shaving practices: three involved shaving (48, 56, 60), three did not involve shaving (50, 51, 58), and one involved only combing (31). The gels used included hydrogels, cryogels, conductive gels, ultrasonic gels, ultrasonic transmission gels, and coupling agents. Detailed information is provided in Table 7.

**Table 7:** TUS device specific information.

**Manufacturer**	**Transducer type**	**FF**	**Targeting method**	**Transducer**	**Shave**	**Gel**	**References**
**1) Focused transcranial ultrasound stimulation** **Custom-made**							
/	Single-element	548 Hz	Neuronavigation	Spherically—focused air—backed piezoceramic transducer	NR	Used, type not mentioned	(55)
	Multi-element	400 kHz	TMS Navigator 3.3	NR	NR	NR	(32)
		650 kHz	MRI-guided	Two spherical phased array transducers	NR	Hydrogel	(49)
				Multiple transducer elements	No	NR	(51)
	Diadem	650 kHz	MRI-guided	NR	No	Cryogel	(50)
**Commercialized**							
Neurosona CoLtd.	NS-US100	250 kHz	MRI and CT-guided	FUS transducer	NR	Cryogel	(20, 21)
				NR	Combed away	Hydrogel	(31)
				NR	No	NR	(58)
Thync Inc.	U+	500 kHz	EEG site F8 MRI and CT-guided	NR	NR	NR	(29)
Brain Industries Inc.	BX Pulsar	650 kHz	MRI-guided and AntNeuro Visor2™	NR	NR	NR	(47, 59)
			MRI-guided	Single-element transducer	Yes	Ultrasonic gel (aquasonic)	(34)
				Spherically focused piezo element	NR	Ultrasound conducting gel—pad (Pharmaceutical Innovations, Newark, NJ)	(54)
Insightec Ltd.	Exablate Neuro	220 kHz	MRI-guided	Transducer helmet array consisting of >1,000 ultrasound transducers	NR	NR	(57)
		650 kHz		15 cm radius hemispherical 1,024 element array	Yes	NR	(48)
NaviFUS Corp.	NaviFUS	NR	Neuronavigation	The specific type of the transducer was not mentioned.	Yes	Used, type not mentioned	(56)
Shengxiang Technology	838B-M-C-II	NR	NR	NR	NR	NR	(41)
**2) Unfocused ultrasound devices** **Custom-made** **LIPUS**							
/	Specially designed convex probe	500 kHz	NR	Convex transducer	NR	NR	(22)
**Commercialized** **dUS**							
Compumed-ics Germany GmbH	DWL Doppler Box X	2.0 MHz	MRI Doppler guidance	NR	NR	NR	(19)
General Electric	LOGIQe	8.0 MHz	The right frontal cerebral cortex	12L-RS probe	NR	Aquasonic 100 ultrasound transmission gel	(45)
Shijiazhuang	DK-102C	800 kHz (± 80)	Transcranial ultrasound headgear	NR	NR	Coupling agent	(46)
**tbTUS**							
Sonic Concepts Inc.	H-246	500 kHz	TMS hot spot	Two element annular array ultrasound transducer	NR	Conductive gel	(44, 52)
**3) Transcranial pulse stimulation**							
Storz Medical	NEUROLITH	4 Hz	MRI-guided	Mobile single transducer	NR	NR	(42, 53)
			MRI-neuronavigation guided	Mobile single transducer	NR	NR	(25, 28)
			MRI-guided and BodyTrack system	Mobile single transducer	NR	NR	(30, 43)
		4 ~ 5 Hz	MRI-guided	Mobile single transducer	NR	NR	(33)
		5 Hz	MRI-guided	Mobile single transducer	NR	Bubble free ultrasound gel	(24)
				NR	NR	NR	(23, 27)
			3D navigation	NR	NR	NR	(26)

### 3.8 Adverse reactions

Twelve studies have reported adverse reactions, including headache (24, 30, 42, 43, 45, 53), fatigue (44, 53), nausea and vomiting (25, 30), naming and memory impairment (56), swelling and scalp fever (56, 57), worsening mood (24), drowsiness (25), pain (25). However, all of these adverse reactions were transient nature and mild in severity.

### 3.9 Regression results

The regression model exhibited an *R*^2^ of 0.420, indicating moderate explanatory power. However, the overall model fit, assessed by an F-test, resulted in a *p*-value of 0.00466. Among the stimulation parameters, the regression coefficients revealed that frequency had a significant negative effect (β = −2.3916, *p* = 0.045), pulse repetition frequency also showed a substantial negative influence (β = −18.2634, *p* = 0.043), and mechanical index exhibited a significant positive impact (β = 38.3535, *p* = 0.044). Conversely, peak negative pressure, spatial-peak pulse-average intensity, duty cycle, and pulse duration did not achieve statistical significance in their effects. The distribution of residuals was normally distributed, and the Durbin-Watson statistic of 2.743 indicated no significant autocorrelation issues within the model. After conducting an in-depth analysis of the interaction of each parameter, it was found that the test results of all interaction terms did not reach the statistically significant level (*p* > 0.05). This indicates that the mutual influence among various parameters is relatively weak and fails to present a synergy effect that has a substantive impact on the research results. For details in Table 8.

**Table 8:** Regression results.

**Variable**	**Coefficient**	**Std. Error**	***t***	**p**	**95% CI lower**	**95% CI upper**
Intercept	0.621	0.352	1.764	0.108	−0.163	1.404
FF	−2.392	1.047	−2.284	0.045^*^	−4.724	−0.059
PNP	172.685	77.978	2.215	0.051	−1.061	346.431
I_SPPA_	−181.261	81.622	−2.221	0.051	−363.127	0.605
PRF	−18.263	7.903	−2.311	0.043^*^	−35.873	−0.654
DC	−32.939	14.896	−2.211	0.051	−66.131	0.252
PD	−20.557	8.981	−2.289	0.045^*^	−40.567	−0.547
MI	38.353	16.683	2.299	0.044^*^	1.180	75.526

^*^p < 0.05.

## 4 Discussion

TUS is a novel non-invasive neuromodulation technique with promising efficacy and potential clinical applications. However, uncertainty in parameter selection greatly limits its promotion and application in clinical practice (61). This study explored the relationship between TUS stimulation parameters and treatment outcomes using a systematic review and multiple linear regression analysis. This study explored the relationship between TUS stimulation parameters and treatment outcomes using a systematic review and multiple linear regression analysis. The systematic review revealed that variations in TUS parameter settings influence treatment efficacy and that different brain regions often require distinct TUS parameters for optimal stimulation. Furthermore, it was observed that the TUS parameters chosen for treating different diseases exhibit unique characteristics tailored to specific conditions. As the current study is in the initial stage, the focus is not strong and the inter-study heterogeneity is large, making it difficult to conduct meta-analysis. Therefore, we tried to use multiple linear regression analysis to explore the relationship between TUS parameters and human physiological response. In addition, our study focused on patient populations, excluding healthy individuals, with a special emphasis on dose-response relationships to better understand the impact of TUS parameters. This approach aims to provide a more robust foundation for future research and clinical applications of TUS. The systematic review revealed that variations in TUS parameter settings influence treatment efficacy and that different brain regions often require distinct TUS parameters for optimal stimulation. Furthermore, it was observed that the TUS parameters chosen for treating different diseases exhibit unique characteristics tailored to specific conditions.

### 4.1 Different stimulation parameters produce different therapeutic effects

The efficacy of TUS is closely related to its parameter settings, including FF, intensity, DC, PRF, and others. TUS can operate in two acoustic paradigms: continuous or pulsed, with the pulsed paradigm being the most used in neuromodulation; specific paradigm settings are detailed in previous reviews (18). Studies show that TUS parameters significantly influence the targeting and effectiveness of ultrasound stimulation of specific brain regions, and different frequency parameters have varying therapeutic effects on different diseases and brain regions.

#### 4.1.1 Fundamental frequency

Fundamental frequency is a key determinant of TUS efficacy, influencing both penetration depth and spatial resolution (62). Low-frequency ultrasound penetrates tissues more easily (63, 64). Although the penetration depth of high-frequency ultrasound is shallower, it offers greater focusing accuracy (65). This is because low-frequency ultrasound has a longer wavelength, lower scattering, and less energy attenuation, whereas the energy attenuation of ultrasound increases with frequency (66). Therefore, low-frequency TUS is generally considered more suitable for stimulating deep brain regions, while high-frequency TUS is appropriate for the cerebral cortex.

Consequently, ultrasound frequencies from 200 to 800 kHz are currently used in fTUS (67), while frequencies from 1 to 15 MHz are used in dUS (68). This study found that ultrasound frequencies of 500 to 650 kHz are used for most cerebral cortex stimulation, while a wider range of frequencies is used for deep brain regions. We speculate that this is due to the following reasons. First, high-frequency ultrasound has a shorter wavelength, enabling accurate energy focusing and thus higher spatial resolution (69, 70). For complex and small nuclei in deep brain areas, such as the amygdala and nucleus accumbens (56, 57, 59), high-frequency TUS can stimulate these regions more precisely, reducing the impact on surrounding non-target tissues and achieving more precise neuromodulation or treatment. Second, high-frequency TUS can reduce thermal effects. Although low-frequency ultrasound penetrates deeper, it requires higher energy, increasing the potential risk of tissue heating. For example, when stimulating the thalamus, high-frequency TUS can achieve effective stimulation at lower power, reducing the risk of thermal damage to surrounding tissues and improving the safety of stimulation (61). Therefore, it is necessary to flexibly adjust the frequency parameters of TUS according to specific research objectives and stimulation areas to achieve accurate and effective neural regulation.

For diagnostic ultrasound, the fundamental frequency ranges from 2 to 8 MHz, providing high-resolution images that help to clearly observe the internal structure of the brain. However, due to the strong attenuation effect of the skull on high-frequency ultrasound, the sound energy level is greatly reduced. Therefore, when using dUS, it is necessary to fully consider the relationship between frequency and energy loss and explore appropriate compensation or optimization methods to improve the accuracy and reliability of TUS.

#### 4.1.2 Intensity

The spatial peak pulse-average intensity (I_SPPA_), spatial peak temporal-average intensity (I_SPTA_), and other intensity indicators determine the degree of ultrasound energy transfer to brain tissue (71). In an animal study, high-intensity fTUS was found to increase motor cortex excitability in rats (72). Previous systematic reviews have suggested that too low ultrasound intensity is insufficient to exhibit neuromodulatory effects (36). Considering the attenuation by the skull, a higher sound intensity is recommended for human studies (73). However, higher intensity does not necessarily yield better therapeutic effects. Increased ultrasound intensity leads to higher tissue temperatures, potentially causing tissue necrosis and irreversible consequences, and increasing the risk of mechanical and thermal effects (74–77). At low intensities, tissue temperature changes are minimal and within the normal physiological range (63, 78). It has been suggested that even small changes in temperature may have significant effects on neural function (79, 80); therefore, the effects of slight temperature changes in the spatially restricted volume of the nervous system need to be explored.

This study found that the sound intensity was lower when applied to cortical regions and higher when applied to deep brain areas. Higher ultrasound intensity enhances penetration and can affect deeper tissues, consistent with the findings of Tufail et al. (63). Therefore, lower sound intensity is recommended for cortical brain regions and higher sound intensity for deep brain regions. However, it is important to adhere to the maximum sound intensity limits proposed by the FDA and ITRUSST, which recommend that I_SPTA_ should be below 94 mW/cm^2^ and I_SPPA_ should be below 190 W/cm^2^ (67, 81).

#### 4.1.3 Other parameters

In addition, parameters such as PD, PRF, DC, SD, and MI greatly affect the mode of brain stimulation and the final therapeutic effect.

Pulse duration varied among the included studies. In epilepsy studies, pulse durations ranged from 0.2 ms to 10 ms (47, 48, 51). PD affects the duration of ultrasound action on neurons. A short pulse duration may only transiently stimulate the neuronal membrane, causing local electrophysiological changes (82). In contrast, a longer pulse duration may maintain the neuron in a continuous stimulation state, triggering more ion channels to open or close, thereby affecting neuronal excitability and neurotransmitter release more profoundly.

The PRF determines the number of ultrasound pulses per unit time. A higher PRF indicates that neurons will be stimulated multiple times in a short period, which may shift the activation-inhibition balance toward excitation and enhance neural activity (83). However, if the PRF is too high, it may lead to excessive excitation or even neuronal damage (84). In contrast, a lower PRF provides a more moderate stimulus, allowing sufficient time for neurons to return to the resting state. This slow and sustained stimulation pattern may be more conducive to regulating damaged neural circuits in chronic diseases, such as Alzheimer's disease (20, 21), promoting neuroplasticity repair without excessive burden on neurons.

The duty cycle refers to the ratio of pulse duration to pulse period, determining the temporal distribution of ultrasound energy. Computational models suggest that low DC inhibits neurons, while high DC activates neurons. However, existing studies lack experiments using consistent outcome measures to compare different DC protocols, necessitating further systematic research (11).

The mechanical index is an important parameter measuring the non-thermal effects of ultrasound, reflecting the strength of the mechanical effects produced during ultrasound propagation. Appropriate mechanical parameters are essential to ensure the efficacy and safety of ultrasound stimulation. If the MI is too high, it may cause unnecessary mechanical damage to brain tissue, such as microhemorrhage or neuronal structural damage (85). Different brain regions and diseases may have different tolerance ranges and optimal MI values, which need to be determined through further research. The FDA recommends that MI be <1.9 (67).

#### 4.1.4 TPS

TPS is a new NIBS technique that differs from traditional fTUS by using ultrasonic pressure pulses to regulate brain activity (24, 86). The main stimulation parameters of TPS (NEUROLITH, Storz Medical) include pulse frequency, pulse width, pulse intensity, and stimulation time. The pulse frequency is usually in the lower range of 1 ~ 5 Hz, using a single ultra-short ultrasound pulse (3 μs), with an energy level of 0.2 ~ 0.25 mJ/mm^2^, and a pulse number ranging from 800 to 6,000 pulses. The TPS system has a lower pulse rate, better skull penetration, and precise localization of specific brain regions, including deep brain regions (26).

Some studies used TPS to treat the anterior part of the brain (EL = 0.2 ~ 0.25 mJ/mm^2^, FF = 4 ~ 5 Hz, 6,000 pulses) for 3 to 4 weeks. Beisteiner et al. found that after 2 weeks of TPS stimulation, cognitive improvement lasted for 3 months (24); Popescu et al. explored the effect of TUS in reducing cortical atrophy in patients with Alzheimer's disease, observing alleviation of cortical atrophy, which shows the potential of TPS to reverse pathological progression in neurodegenerative diseases (23). For adolescents with ASD and ADHD, improvement was shown after stimulation of relevant regions with TPS (42, 43). Unlike the other studies, these two studies used 800 pulses for treatment, possibly because the patients were teenagers, suggesting that different ages may have their own suitable frequency ranges.

#### 4.1.5 TUS induce LTP-like plasticity and LTD-like plasticity

Like TMS and tDCS (87), transcranial ultrasound stimulation holds the potential to induce neural plasticity within the human brain. Bao's study showed that TUS can trigger both long-term potentiation (LTP)—like and long-term depression (LTD)—like plasticity in the human brain (88). The underlying mechanism of TUS—induced LTP—like plasticity may be associated with the activation of voltage—gated ion channels (89). When these channels are activated, it leads to an enhancement in synaptic efficacy. Specifically, the mechanical vibrations generated by TUS could promote the release of excitatory neurotransmitters such as glutamate (90). This, in turn, boosts long—lasting enhanced excitability, closely resembling the LTP process in the brain. Research on the cellular effects of ultrasound has shown that it can influence ion channels and neurotransmitter release, providing a basis for this proposed mechanism (91).

In a study by Clennell et al., low-intensity TUS applied to rat cortical primary neurons at a specific frequency of 200 kHz resulted in an increase in the average frequency of evoked action potentials, indicating enhanced neuronal excitability, like the effect of LTP-like plasticity (92). In a study by Zhang et al., researchers found that low-intensity TUS applied to the motor cortex at a specific frequency of 200 kHz had an inhibitory effect on neuronal activity, as seen in a reduction in the amplitude of motor evoked potentials, and the results suggest that this low-intensity TUS can modulate cortical excitability (93).

### 4.2 Effects of TUS in the treatment of various brain regions

The physiological function and pathological characteristics of different brain regions affect the choice of TUS parameters (94). In clinical treatment, understanding the relationship between TUS parameters and brain regions is helpful for different diseases, and existing research parameters can be referred to select the appropriate frequency and intensity to improve the effectiveness and safety of treatment.

#### 4.2.1 Hippocampus

In the study of the hippocampus, the combination of parameters used to treat AD included a frequency of 250 kHz, I_SPPA_ is 0.5 ~ 3 W/cm^2^, PRF is 2 Hz and DC is 4%. This parameter setting resulted in slight improvements in memory, executive function, and cognition (20, 21). The hippocampus is primarily responsible for consolidating short-term memories into long-term memories, playing a key role in spatial navigation, learning, and cognition (95). The cavitation effect of low-frequency TUS is weak, and it primarily induces inhibition of overexcited or abnormally firing neurons through mechanical vibration, thus alleviating symptoms (82). Meanwhile, we found that although both the amygdala and hippocampus are involved in neural activities related to emotion regulation, TUS parameters were different in studies on them. The amygdala plays an important role in emotion processing, especially in neural circuits related to anxiety (96). TUS with higher frequency and specific intensity may alleviate anxiety symptoms by regulating the excitability of the amygdala (59). However, the hippocampus plays a prominent role in long-term emotional memory, and its parameter settings focus more on influencing long-term neuronal plasticity.

During epileptic seizures, the hippocampus is one of the important brain regions involved (97). TUS can regulate the membrane potential and ion channel activity of neurons, restoring neuronal excitability to normal levels and thus reducing the frequency and severity of seizures (98). It is a potential adjuvant therapy for DRE.

#### 4.2.2 Thalamus

In thalamic studies, the combination of parameters used to treat ET included a frequency of 650 kHz, a PRF of 10 ~ 167 Hz, and a DC of 5 ~ 33.4 %, resulting in reduced tremor symptoms and improved ability to perform daily activities (47, 48, 51). The thalamus is an important hub for sensory transduction, transferring sensory information (except smell) to the cerebral cortex, and is also involved in the regulation of consciousness, sleep-wake cycles, and motor control (99). TUS can modulate neuronal activity and reduce neuronal hyperexcitability in patients with epilepsy (98).

#### 4.2.3 Anterior cingulate cortex

The ACC plays a key role in emotion regulation, cognitive control (100), and pain perception (101). Like transcranial magnetic stimulation, transcranial ultrasound also has high frequency excitation and low frequency inhibition (102). In pain-related diseases, 250 kHz can inhibit the excitability of neurons in pain-related brain areas, regulate the transmission and perception of pain signals, and thus relieve pain symptoms (58). In depression-related TRD research, 650 kHz can alleviate depressive symptoms by improving the excitability of neurons in depression-related brain regions (50). TUS of specific frequency and intensity alters neural activity in emotion, cognition, and pain perception by affecting levels of neurotransmitters such as serotonin and dopamine (103) or the membrane potential of neurons (104).

#### 4.2.4 Motor cortex

The motor cortex is an important region responsible for the control and regulation of motor function (105). For motor cortical areas, tbTUS with a frequency of 500 kHz was used in PD treatment studies (44, 52) to act on ion channels and synapses of motor neurons, altering neuronal excitability and synaptic transmission efficiency (106). By regulating the activity of calcium channels, it affects neural signal transduction related to neurotransmitter release and muscle contraction, thereby increasing the excitability of the motor cortex, and improving motor function (63).

#### 4.2.5 Frontal cortex

The frontal cortex is involved in higher cognitive functions and voluntary motor control and is associated with emotional expression and personality (107). In MDD studies, parameters such as 250 kHz frequency, 1 ms pulse duration, 5 Hz pulse repetition rate, and 50% duty cycle were used to improve MADRS scores and reduce depression (31). In contrast, Schachtner et al. (32), used different frequency parameters (400 kHz) and produced positive effects on depression, suggesting that there may be an effective frequency range in the treatment of depression in the frontal cortex. Different frequencies within this range improve depressive symptoms by modulating neural circuits associated with emotion regulation (108). Other parameters such as pulse duration, pulse repetition rate, and duty cycle also play important roles in their respective studies. They cooperate with frequency parameters to affect neural activity in the frontal cortex, thereby reducing depressive symptoms. Future research needs to explore the optimal parameter combinations in more depth.

#### 4.2.6 Temporal lobe

The temporal lobe is primarily responsible for processing auditory information and plays an important role in memory, emotion, language comprehension, and visual perception (109). In ET studies, a frequency of 650 kHz, PD of 10 ms, PRF of 100 Hz, and DC of 10 % were used to stimulate the temporal cortex, resulting in reduced tremor amplitude (47, 48, 51). This suggests that with this combination of parameters, ultrasound can exert a positive effect on the neural circuitry of the temporal cortex related to motor control. The 650 kHz frequency may have a resonance effect with the natural frequencies of specific neuronal populations within the temporal cortex, enhancing the synchronous activity of these neurons (110). In DRE studies, the RAVLT showed a slight decrease under parameters of 650 kHz frequency, 0.2 ms pulse duration, 250 Hz pulse repetition rate, and 50 % duty cycle. However, when the parameters were changed to 650 kHz, 0.5 ms pulse duration, 100 Hz pulse repetition rate, and 5 % duty cycle, different results appeared (54–56). This indicates that different combinations of parameters have different effects on cognitive function in the temporal cortex. High-frequency short pulses and high duty cycles may interfere with normal information interaction between the temporal cortex and memory-related brain areas, affecting memory function evaluations like the RAVLT.

### 4.3 Effects of TUS in the treatment of various functional disorders

#### 4.3.1 Improvement in cognitive function

Through a systematic review of the included studies, this paper found that TUS has a significant effect on neurodegenerative diseases, especially in the studies of AD, where ultrasound stimulation parameters show specific patterns and effects. AD, one of the major neurodegenerative diseases, is often accompanied by cognitive decline and memory impairment (111). By activating ion channels on neuronal cell membranes and enhancing neuroplasticity (112).

From the frequency perspective, the range of 250 to 500 kHz has shown effectiveness in different studies. Jeong et al. used 250 kHz fTUS to stimulate the hippocampus of AD patients, resulting in improved memory, executive function, and cognitive ability without opening the blood–brain barrier (20, 21). This may be due to the ability of this frequency to promote the regulation of neural signaling and synaptic plasticity in hippocampal neurons (113). In terms of intensity, an I_SPPA_ range of 0.5 ~ 3 W/cm^2^ has a positive effect. The appropriate intensity ensures that ultrasound energy is sufficient to activate neurons without causing brain tissue damage (80). Ultrasound stimulation at this intensity may improve cognitive function in AD patients by regulating biochemical processes within neurons, such as promoting neurotransmitter release or modulating ion channel activity. Additionally, studies by Shimokawa et al. (22), using LIPUS, and by Nicodemus et al. (19), using dUS, further confirmed the efficacy of ultrasound stimulation for AD from different perspectives. Despite slight differences in parameters, both studies showed the potential of ultrasound in treating neurodegenerative diseases.

Based on the above studies, frequency range 250 ~ 500 kHz, intensity with I_SPPA_ of 0.5 ~ 3 W/cm^2^, and for PRF and DC, using PRF of 20 Hz and DC of 4 % to stimulate the hippocampus has a potential positive effect on AD patients.

#### 4.3.2 Improvement of movement disorders

In movement disorders, TUS has a significant effect on improving motion-related symptoms, and ultrasound parameters have their own characteristics in different disease types.

In the essential tremor study, the fundamental frequency of 650 kHz was used in all three studies, indicating that this frequency may be critical for ET treatment. Bancel et al. (48) and Deveney et al. (47) stimulated the VIM with specific parameters, reducing tremor and improving the ability to perform activities of daily living. Similar positive results were obtained by Riis et al. (51) when stimulating the anterior medial thalamic nucleus (A-MTL). This indicates that different parameter combinations based on a frequency of 650 kHz affect the neural circuit related to tremor by regulating neuronal activity in VIM, A-MTL, and other brain areas, thereby improving motor symptoms.

In Parkinson's disease studies, tbTUS at 500 kHz has shown positive effects on motor cortical areas. However, the changes of UPDRS-III scores in patients were different in different studies, such as Grippe et al. (52) and Osou et al. (53) showed a decrease in the score, while Samuels et al. (44) showed no significant change, which may be related to differences in sound intensity. This indicates that in the treatment of movement disorders, intensity parameters have an important impact on therapeutic effects. Appropriate intensity can more effectively regulate the excitability of neurons in the motor cortex, optimizing neural signal transmission related to motor control and thus improving motor function (80). At the same time, the PRF (5 ~10 Hz) and DC (5 ~ 10 %) within certain ranges also contribute positively to the treatment effect, acting together on motor-related brain areas to regulate neuronal firing patterns and neural circuit stability (61).

Based on the above studies, frequency range of 500 ~ 650 kHz, intensity with I_SPPA_ of 14.39 ~ 20 W/cm^2^, PRF of 5 ~ 10 Hz and DC of 5 ~ 10 % has a potential positive effect on AD patients.

#### 4.3.3 Alleviation of mental disorders and mental disorders

In the field of psychiatric and psychological disorders, ultrasound stimulation parameters have been shown to be effective in relieving symptoms under different disease types and stimulation targets.

In the studies on the treatment of MDD, TRD, trGAD, and SUD with TUS, different frequencies of ultrasound stimulation to different prefrontal cortex or other relevant brain area targets have improved mood and related symptoms. For example, Oh et al. (31), Schachtner et al. (32), and Reznik et al. (29), used 250 kHz, 400 kHz, and 500 kHz to stimulate different prefrontal cortex targets to treat MDD, and Riis et al. (49, 50), used 650 kHz to stimulate different brain regions to treat TRD, all finding improvements in depressive symptoms. Mahdavi et al. (59) used stimulation of the right amygdala at 650 kHz to treat trGAD and reduced anxiety symptoms. Mahoney et al. (57) used 220 kHz frequency to stimulate the Nac of patients with SUD, reducing substance craving. TUS may regulate neural activity in brain areas related to emotion regulation by affecting on neurotransmitter systems such as serotonin and dopamine, thereby improving the emotional state and psychological symptoms (110).

Zhang et al. applied 500 kHz rTUS to stimulate the left primary motor cortex (l-M1) in healthy individuals and found long-lasting excitatory effects of ipsilateral M1 lasting up to 30 min, suggesting that TUS can enhance LTP-like plasticity in the long term and improve cognitive function (114). Another study by the same team also found that TUS improves excitability while regulating the interhemispheric balance of bilateral M1 excitability for up to 30 min, suggesting that rTUS has considerable potential for brain disease (115). In addition, in a double-blind randomized, pseudo-controlled study of rTUS in patients with schizophrenia, 15 doses of excitatory rTUS on the left DLPFC were found to alleviate negative symptoms and improve cognitive performance in patients with schizophrenia, suggesting that rTUS has considerable potential for clinical use (116).

Based on the above studies, the combination of FF ranging from 400 to 650 kHz, I_SPPA_ ranging from 14.39 to 31 W/cm^2^, pulse repetition rate around 5 Hz, and duty cycle 5 ~ 8% has a potential positive effect on mental and psychological disorders.

#### 4.3.4 Relief of symptoms of other central nervous system disease

In other central nervous system diseases, including stroke and epilepsy, ultrasound stimulation parameters have an important impact on treatment and symptom relief.

In stroke studies, Wang et al. (41) used fTUS to stimulate the FC and Lu YH et al. (46) used TUS to stimulate the affected area, both showing positive effects on cognitive function, pain relief, and limb motor function. The results indicate that ultrasound stimulation in the frequency range of 500 ~ 650 kHz can affect functions of brain regions related to cognition, movement, and pain perception by repairing and regulating damaged neural circuits after stroke, promoting neuroplasticity, and improving regional cerebral blood flow (117). In epilepsy studies, Lee, Stern, and Brinker et al. (54–56) used fTUS to treat DRE patients, and different stimulation parameters all showed a reduction in seizure frequency but suggested a possible impact on cognitive function. For example, a mild decrease in the Rey Auditory Verbal Learning Test (RAVLT) was found in the study by Stern et al. It is suggested that ultrasound stimulation parameters should be carefully selected, paying attention to seizure control and cognitive function protection.

Based on the above studies, frequency range of 500 ~ 650 kHz and I_SPPA_ below 2.8 W/cm^2^ has a potential positive effect on mental and psychological disorders.

#### 4.3.5 Treatment of pain disease

In the treatment of pain diseases, ultrasound stimulation also shows a positive effect. Hameroff et al. (45) used dUS to stimulate the PFC of patients with chronic pain, and Shin et al. (58) used fTUS to stimulate the ACC of patients with chronic neuropathic pain, both relieving pain symptoms and improving patient mood.

These recommended parameters are based on different studies, and different TUS techniques (such as fTUS, LIPUS, dUS, TPS) may need to be further optimized and adjusted according to the actual situation in the application. At the same time, safety and individual differences among patients should be fully considered. It should be noted that these parameters need to be adjusted and optimized due to different TUS techniques, and since the research is still in the exploratory stage, safety and individual patient differences should be fully considered.

### 4.4 Other influencing factors

In the research and application of ultrasonic stimulation, in addition to TUS parameters, positioning and navigation methods, transducer characteristics, gel type, and hair shaving practices all have important effects on TUS efficacy.

The application of positioning and navigation technology reflects the importance of accurately determining the target tissue position in ultrasound therapy. Various positioning and navigation methods were involved in the included studies. MRI navigation has the advantages of high resolution and clear soft tissue imaging, providing accurate anatomical information for the positioning of ultrasound transducers (60, 118–120). Combining MRI with other techniques, such as TMS (121, 122), and EEG (123, 124), can further improve localization accuracy and comprehensiveness, helping to understand the relationship between brain electrophysiological activity and the target area.

The transducer plays a central role in the TUS device, and its shape and arrangement affect the focusing effect of ultrasound (125). Focused ultrasound devices include spherical phased-array transducers and spherical focusing piezoelectric elements, which can precisely focus ultrasound on specific areas. Non-focused ultrasound transducers generally mean that the transducers with flat but not concave structures, which are more suitable for stimulating large areas of superficial tissues or regions with lower accuracy requirements. (35, 37). In clinical application, it is necessary to select the transducer type reasonably according to the treatment goal and tissue characteristics to achieve the best therapeutic effect.

In the included studies, only three reported shaving patients' hair. Previous studies have argued that although hair is seen as a potential obstacle in TUS treatment, the ultrasound wavelength is relatively large compared to hair thickness when using low-frequency TUS, and the loss of acoustic energy at the focus is negligible (37). This indicates that it may be possible to decide whether patients need to shave based on ultrasound intensity and frequency in TUS studies, but further research is needed to verify applicability in different diseases.

Ultrasound gels are often used as acoustic couplers to facilitate sound wave transmission in TUS treatment. The presence of a coupling medium can effectively reduce the reflection of acoustic waves at the interface and improve the efficiency of ultrasonic energy transmission to the cerebral cortex or deep brain areas. Different coupling media have different acoustic properties. For example, hydrogels have good compressibility and skin conformity, better filling the small gap between the transducer and the scalp to ensure stable sound wave transmission (126, 127). Cryogels may have unique applications in specific cryogenic ultrasound therapy scenarios (128, 129). The diversity of gel types provides more options, but further research is needed on the optimal application of each gel under different ultrasound parameters, disease types, and treatment conditions to fully exploit their advantages and improve ultrasound treatment quality.

### 4.5 Adverse reactions

The incidence and types of adverse effects varied across TUS studies, and most adverse effects were transient and mild. Headache was the most common adverse reaction, but most cases resolved spontaneously or shortly after treatment without special intervention. Other adverse effects such as fatigue, nausea, vomiting, mood deterioration, and drowsiness were also mostly transient (duration: <24 hours). No permanent side effects were reported.

### 4.6 Limitation

Although this study has systematically summarized the relationship between research parameters and effects in the existing field of transcranial focused ultrasound, there are still some limitations; (1) Due to the high heterogeneity of research designs, result reporting methods, and statistical data, it is impossible to conduct unified effect size calculations and weighted combinations. To a certain extent, this restricts the quantitative comparison of results and the clear definition of the overall effect, only providing a systematic description and induction; (2) Although this study attempts to explore the relationship between ultrasound parameters and effects through multiple regression analysis. However, the linear relationship assumed by the regression analysis may not fully reflect the complex non-linear association between ultrasound parameters and effects; (3) Currently, the overall literature in the field of transcranial focused ultrasound is relatively limited, with problems such as insufficient sample sizes, imperfect designs, and non-standardized reports, further limiting the reliable assessment of effects. (4) This study analyzed only English and Chinese literature, which may limit the generalizability and representativeness of the findings. Future studies can be further expanded to the literature in other languages to evaluate the efficacy and parametric optimization of TUS more fully. Therefore, the regression analysis results of this study should be interpreted with caution and are more about providing hypotheses rather than conclusive confirmatory conclusions.

### 4.7 Challenges in clinical application and future research directions

Despite the promising potential of TUS for treating various neurological and psychiatric disorders, several challenges hinder its clinical adoption. First, there is a lack of standardization in TUS equipment, parameters, and operational protocols across different studies, leading to poor comparability of results. To enhance reliability and generalizability, future research should focus on conducting multicenter randomized controlled trials that adhere to consistent parameter standards. Second, individual physiological differences among patients may significantly influence TUS efficacy. Future studies should investigate how factors such as age, gender, and disease progression affect TUS outcomes. Stratified analyses could help identify optimal parameter settings tailored to different patient profiles. Lastly, advancements in navigation and localization technologies—such as the multimodal integration of MRI, CT, and EEG—can enhance the precision of targeting specific brain regions, thereby improving the efficacy of TUS treatments.

## 5 Conclusion

This study systematically investigated the effects of TUS parameters, including fundamental frequency (FF), pulse repetition frequency (PRF), pulse duration (PD), and mechanical index (MI), on treatment outcomes. The findings indicate that parameter selection is closely related to the target brain region. For example, low-frequency TUS (200–500 kHz) is more suitable for deep brain stimulation, while high-frequency TUS (500–650 kHz) is most effective for cortical regions.

Additionally, TUS parameters should be adjusted according to the specific pathophysiology and functional impairments of each disorder. For instance, in neurodegenerative diseases such as Alzheimer's, a frequency range of 250–500 kHz and an I_SPPA_ of 0.5–3 W/cm^2^ have a positive impact on cognitive function. For movement disorders like essential tremor, frequencies of 500–650 kHz and I_SPPA_ values of 14.39–20 W/cm^2^ are effective in alleviating symptoms. Future studies should focus on exploring the dose-response relationships of TUS parameters and their long-term effects on neuroplasticity and disease modification, which will be critical for advancing its clinical application.

## Data Availability

The datasets presented in this study can be found in online repositories. The names of the repository/repositories and accession number(s) can be found in the article/Supplementary material.
