# Supplementary material for: Transcranial ultrasound stimulation parameters for neurological diseases: a systematic review
Source: Front Neurol. 2025 May 21;16:1567482. doi: 10.3389/fneur.2025.1567482 (PMC12135807; doi:10.3389/fneur.2025.1567482)
Supplement: Supplementary file 1 [file Table_1.docx]

**Supplement table 1. Web of Science Search Strategy**

| Step | Retrievable |
| --- | --- |
| #1 | "neuromodulation"[Topic] |
| #2 | "transcranial ultrasound"[Topic] OR "transcranial ultrasound stimulation"[Topic] |
| #3 | "focused ultrasound"[Topic] OR "focused ultrasound stimulation"[Topic] |
| #4 | "transcranial focused ultrasound"[Topic] OR “transcranial focused ultrasound stimulation"[Topic] |
| #5 | "transcranial pulse stimulation"[Topic] OR "transcranial unfocused ultrasound stimulation"[Topic] |
| #6 | "transcranial pulse stimulation"[Topic] |
| #7 | #2 OR #3 OR #4 OR #5 OR #6 |
| #8 | #1 AND #7 |

**Supplement table 2. EBSCOhost of Science Search Strategy**

| Step | Retrievable |
| --- | --- |
| #1 | "neuromodulation"[Topic] |
| #2 | "transcranial ultrasound"[Topic] OR "transcranial ultrasound stimulation"[Topic] |
| #3 | "focused ultrasound"[Topic] OR "focused ultrasound stimulation"[Topic] |
| #4 | "transcranial focused ultrasound"[Topic] OR “transcranial focused ultrasound stimulation"[Topic] |
| #5 | "transcranial pulse stimulation"[Topic] OR "transcranial unfocused ultrasound stimulation"[Topic] |
| #6 | "transcranial pulse stimulation"[Topic] |
| #7 | #2 OR #3 OR #4 OR #5 OR #6 |
| #8 | #1 AND #7 |

**Supplement table 3. Cochrane of Science Search Strategy**

| Step | Retrievable |
| --- | --- |
| #1 | "neuromodulation"[Mesh] |
| #2 | "transcranial ultrasound" OR "transcranial ultrasound stimulation" |
| #3 | "focused ultrasound" OR "focused ultrasound stimulation" |
| #4 | "transcranial focused ultrasound" OR “transcranial focused ultrasound stimulation" |
| #5 | "transcranial pulse stimulation" OR "transcranial unfocused ultrasound stimulation" |
| #6 | "transcranial pulse stimulation" |
| #7 | #2 OR #3 OR #4 OR #5 OR #6 |
| #8 | #1 AND #7 |

**Supplement table 4. OVID-Medline of Science Search Strategy**

| Step | Retrievable |
| --- | --- |
| #1 | "neuromodulation"[Mesh] |
| #2 | "transcranial ultrasound" OR "transcranial ultrasound stimulation" |
| #3 | "focused ultrasound" OR "focused ultrasound stimulation" |
| #4 | "transcranial focused ultrasound" OR “transcranial focused ultrasound stimulation" |
| #5 | "transcranial unfocused ultrasound " OR "transcranial unfocused ultrasound stimulation" |
| #6 | "transcranial pulse stimulation" |
| #7 | #2 OR #3 OR #4 OR #5 OR #6 |
| #8 | #1 AND #7 |

**Supplement table 5. Embase of Science Search Strategy**

| Step | Retrievable |
| --- | --- |
| #1 | **'**neuromodulation'/exp OR neuromodulation |
| #2 | 'transcranial ultrasound stimulation'/exp OR 'transcranial ultrasound stimulation' |
| #3 | 'transcranial focused ultrasound':ab,ti OR 'focused ultrasound therapy':ab,ti OR 'transcranial unfocused ultrasound':ab,ti OR 'transcranial pulse stimulation':ab,ti |
| #4 | #2 OR #3 |
| #5 | #1 AND #4 |

**Supplement table 6. Clinical trials of Science Search Strategy**

| Step | Retrievable |
| --- | --- |
| #1 | Transcranial Focused Ultrasound[Transcranial Focused Ultrasound] |

**Supplement table 7. Cnki of Science Search Strategy**

| Step | Retrievable |
| --- | --- |
| #1 | "经颅超声刺激"[主题] |
| #2 | "聚焦超声刺激"[主题] |
| #3 | "经颅聚焦超声"[主题] |
| #7 | #1 OR #2 OR #3 |

**Supplement table 8. Wanfang of Science Search Strategy**

| Step | Retrievable |
| --- | --- |
| #1 | "经颅超声刺激"[全部] |
| #2 | "聚焦超声刺激"[全部] |
| #3 | "经颅聚焦超声"[全部] |
| #7 | #1 OR #2 OR #3 |

**Supplement table 9. VIP of Science Search Strategy**

| Step | Retrievable |
| --- | --- |
| #1 | "经颅超声刺激" |
| #2 | "聚焦超声刺激" |
| #3 | "经颅聚焦超声" |
| #7 | #1 OR #2 OR #3 |
